# Evaluation of microbial and physicochemical properties of water in swimming pools of Guilan province in 2017

**Published:** 2019-07

**Authors:** Mehdi Javahershenas, Alireza Saeedi Jurkuyeh, Mohammad Naeemi Jubneh

**Affiliations:** ^*a*^Department of Environmental Health Engineering, Deputy of Health, Guilan University of Medical Sciences, Rasht, Iran.; ^*b*^Health and Environmental Research Center, Guilan University of Medical Sciences, Guilan, Rasht, Iran.

## Abstract

**Background:**

Water contamination of swimming pools can cause skin and fungal diseases, intestinal disorders, diarrhea and eye and ear diseases in swimmers.

**Methods:**

This descriptive cross-sectional study was carried out in 41 swimming pools in 15 cities of Guilan province in 2017. A total of 505 samples were taken to conduct turbidity tests, residual free chlorine, microbial heterotrophic experiments, and thermoduric coliform in accordance with national and international standards for swimming pools. Statistical analysis was performed by SPSS software (Version 16).

**Results:**

The results showed that 10.8% of water in the swimming pools had no residual free chlorine, 33.1% had undesired turbidity, 9.3% had heterotrophic microbial contamination, and 6.7% had microbial contamination of thermoduric coliforms. This study showed a significant relationship between swimming pool water and the absence of free chlorine, high turbidity, and the presence of heterotrophic microorganisms and thermoduric coliforms (P<0.05).

**Conclusions:**

The present study concluded that the water of swimming pools with low free chlorine and turbidity higher than the standard level has microbial contamination. To prevent water pollution in swimming pools, use of a suitable filtration system to reduce the turbidity of the water, supply of the remaining active disinfection such as chlorine in sufficient quantities (1 to 3 mg/L), compliance with health standards, bathing swimmers before entering the pool, more frequent and timely monitoring by pool operators and environmental health inspectors, especially in peak use times are offered.

**Keywords:**

Swimming Pool; Turbidity; Thermoduric Coliform; Heterotrophic

